# Diaphragmatic motor cortex hyperexcitability in patients with chronic obstructive pulmonary disease

**DOI:** 10.1371/journal.pone.0217886

**Published:** 2019-12-18

**Authors:** Rehab Elnemr, Rania Ahmad Sweed, Hanaa Shafiek

**Affiliations:** 1 Physical Medicine, Rheumatology and Rehabilitation Department, Faculty of Medicine, Alexandria University, Alexandria, Egypt; 2 Chest Diseases Department, Faculty of Medicine, Alexandria University, Alexandria, Egypt; Universita degli Studi di Catania, ITALY

## Abstract

**Background and objectives:**

Respiratory muscles dysfunction has been reported in COPD. Transcranial magnetic stimulation (TMS) has been used for assessing the respiratory corticospinal pathways particularly of diaphragm. We aimed to study the cortico-diaphragmatic motor system changes in COPD using TMS and to correlate the findings with the pulmonary function.

**Methods:**

A case control study recruited 30 stable COPD from the out-patient respiratory clinic of Main Alexandria University hospital- Egypt and 17 healthy control subjects who were subjected to spirometry. Cortical conduction of the diaphragm was performed by TMS to all participants followed by cervical magnetic stimulation of the phrenic nerve roots. Diaphragmatic resting motor threshold (DRMT), cortical motor evoked potential latency (CMEPL), CMEP amplitude (CMEPA), peripheral motor evoked potential latency (PMEPL), PMEP amplitude (PMEPA) and central motor conduction time (CMCT) were measured.

**Results:**

66.7% of COPD patients had severe and very severe COPD with median age of 59 (55–63) years. There was statistically significant bilateral decrease in DRMT, CMEPA and PMEPA in COPD group versus healthy subjects and significant increase in CMEPL and PMEPL (*p* <0.01). Left CMCT was significantly prolonged in COPD group versus healthy subjects (*p* <0.0001) but not right CMCT. Further, there was significant increase in CMEPL and CMCT of left versus right diaphragm in COPD group (*p* = 0.003 and 0.001 respectively) that inversely correlated with FEV_1_% and FVC% predicted. Right and left DRMT were insignificantly different in COPD group (*p* >0.05) but positively correlated with FEV_1_/FVC, FEV_1_% and FVC% predicted.

**Conclusion:**

Central cortico-diaphragmatic motor system is affected in COPD patients with heterogeneity of both sides that is correlated with pulmonary function.

**Significance:**

Coticospinal pathway affection could be a factor for development of diaphragmatic dysfunction in COPD patients accordingly its evaluation could help in personalization of COPD management especially pulmonary rehabilitation programs.

## Introduction

Chronic obstructive pulmonary disease (COPD) is mainly presented with dyspnea and exercise limitation secondary to irreversible airflow obstruction; however, nowadays COPD is considered as multi-systemic inflammatory disorder rather than simple respiratory disease. [1] Respiratory muscles dysfunction, including the diaphragm which is considered the main inspiratory muscle, has been reported in COPD compared to healthy elderly individuals [2] and has been implicated in the development of dyspnea. Diaphragmatic dysfunction in COPD is either mechanically secondary to air trapping and hyperinflation in COPD leads to chronic reduction of the apposition zone of the diaphragm [3] and shorten of the diaphragm fiber sarcomere [4]; or local activation of muscle proteases and oxidative stress due to inspiratory loading induces structural muscular injury. [5,6]

Transcranial magnetic stimulation (TMS) aims at measuring neuronal electrical activity [7] using a stimulation device and a transducing coil. [8] The principle of TMS is based on induction of an electromagnetic field in the brain of sufficient magnitude and density to depolarize the neurons. [9] TMS pulse applied over the primary motor cortex, using the stimulation coil placed tangentially on the head, induces action potentials in cortical axons that spreads trans-synaptically to neurons along the corticospinal tract and peripheral motor nerve. [10,11] These excitation signals elicit responses in those muscles that receive corticomotor input from the stimulated motor cortical area which are recorded as motor evoked potentials (MEPs). [10] TMS has been used as an investigation tool for assessing the respiratory corticospinal pathways and studying of diaphragmatic MEPs. [7,12,13]

In the last decade, a study demonstrated increased excitability of the motor cortex controlling respiratory muscles in COPD especially diaphragm which could be secondary to increased inspiratory load and subsequent elevated respiratory drive. [14] Recently, other studies found dysfunction of the corticospinal motor pathway assessed by TMS during acute exacerbation of COPD patients with or without respiratory failure. [15,16] TMS was also used to assess the response of genioglossus and diaphragm during inspiratory maneuvers in awake patients of obstructive sleep apnea syndrome (OSAS) as well as asleep. [17] Further, increased motor cortex inhibition in OSAS with abnormal plasticity related TMS phenomena has been demonstrated in another study. [18] However, still little research has been conducted in COPD to assess central neural drive to the diaphragm and its possible involvement in physiological derangement in COPD patients. Accordingly, we aimed to study the cortico-diaphragmatic motor system changes in COPD patients using TMS compared to healthy subjects as primary outcome. In addition, we aimed to correlate between the MEPs changes and the pulmonary function; and to detect possible cut-off value for corticospinal diaphragmatic pathway affection that could be a reference in this group of patients as secondary outcomes.

## Material and methods

### Study design and characterization of studied population

A case-control study that recruited stable COPD patients according to GOLD guidelines 2017 [1] attended the out-patient respiratory clinic of Main Alexandria University hospital, Egypt as well as healthy control subjects between February 2017 to April 2018. Fifty-six COPD patients and 30 healthy control subjects were invited to participate in the study, however, only 30 COPD patients and 17 healthy controls accepted to participate and complete all the tests.

All COPD patients were stable i.e. no COPD exacerbation in last 4 weeks, and were proved to have airway obstruction using spirometry (post-bronchodilator FEV_1_/FVC < 0.70) according to GOLD guidelines 2017. [1] The patients were further classified based on GOLD classification into: mild (GOLD 1: FEV_1_ ≥ 80% predicted), moderate (GOLD 2: 50% ≤ FEV_1_ < 80% predicted), severe (GOLD 3: 30% ≤ FEV_1_ < 50% predicted), and very severe disease (GOLD 4: FEV_1_ < 30% predicted). [1] All patients who were known to have COPD exacerbation, current oral corticosteroids therapy or within last 30 days, bronchial asthma, interstitial lung diseases, metabolic diseases (mainly diabetes mellitus, uremia and hepatic failure), neurological diseases (as cerebrovascular stroke, epilepsy, peripheral neuropathy and muscle diseases), body mass index (BMI) more than 40 kg/mm2, history of drug abuse, history of any neoplasm, or any contraindications for magnetic stimulation were excluded from the study. Further, 17 healthy control subjects with normal lung function referred for check-up were recruited from other clinics.

All the participants underwent detailed history taking specifically age, sex, smoking habits, respiratory symptoms, current medications including inhalers, followed by clinical full examination and chest X-ray. Spirometry was performed to all participants according to ATS / ERS guidelines [19] as post- bronchodilator FVC, FEV_1_ and FEV_1_/FVC ratio were recorded; at least 3 acceptable trials were recorded for each participant and the best values were considered. For COPD patients, arterial blood gases (ABG) were assessed for COPD patients, and venous blood sample was taken for measurement of fasting blood glucose, liver function testing, renal function testing, complete blood picture, and serum electrolytes (sodium and potassium). Computed tomography of chest was performed if indicated clinically. The study has been approved by the scientific committee of faculty of medicine, Alexandria University, Egypt, and a written informed consent was obtained from all participants.

### Diaphragmatic neural function assessment

**Fig 1:**
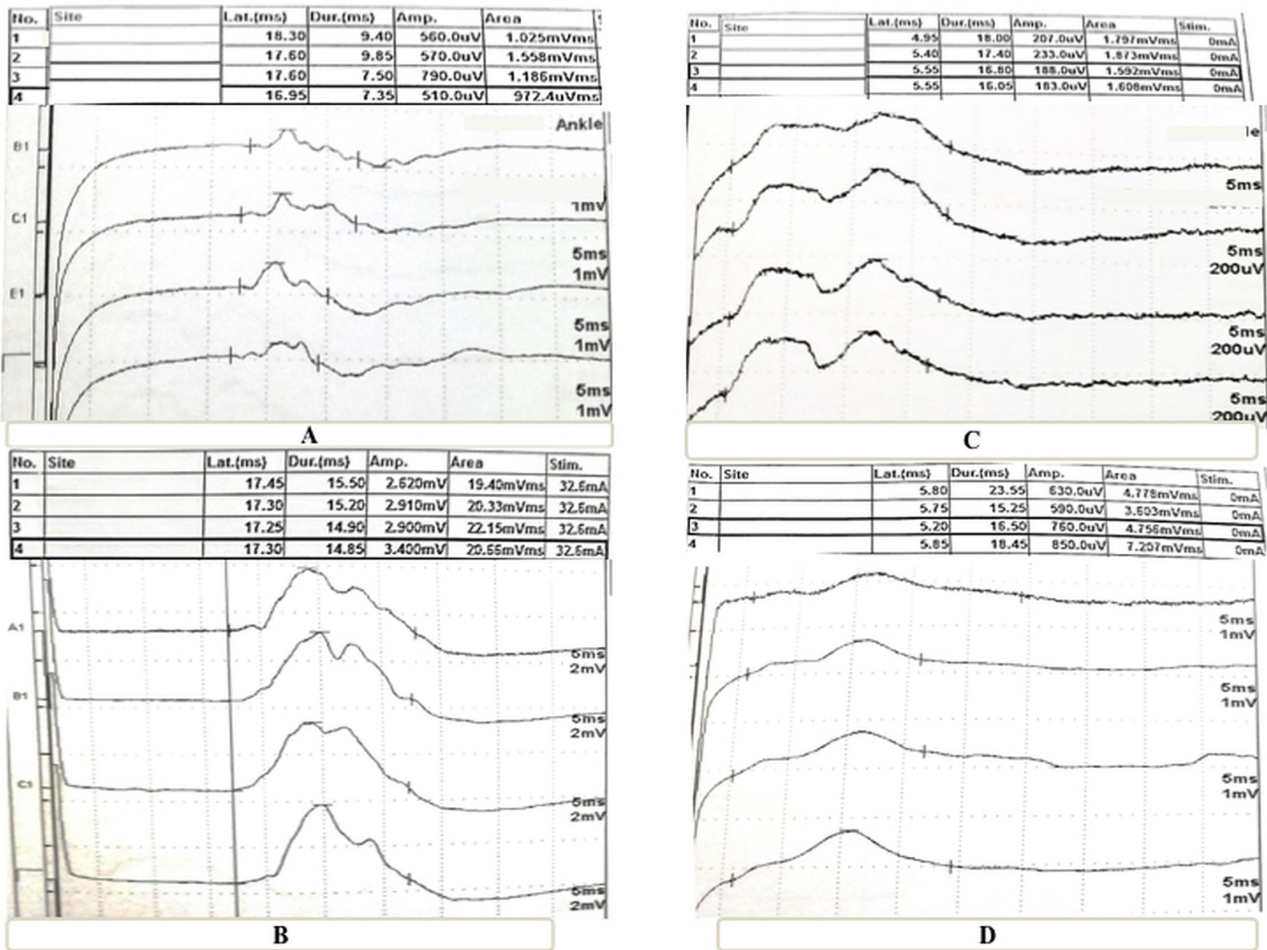
Demonstration example of CMEP and PMEP diaphragmatic signals in COPD and normal Subject. (A) CMEP of diaphragm in COPD patient noticing that there is delayed latency and low amplitude of the response versus Fig 1B which represents healthy subject; (C) PMEP of diaphragm in COPD patient with low amplitude of the response versus Fig 1D which represents healthy subject.

Firstly, TMS of the diaphragm was carried out using Neuropack electrophysiological apparatus (Nihon Kohden MEB-7102K Tokyo, Japan) and a 90-mm circular coil (with peak magnetic field strength of 2 Tesla) that has an outer diameter of 115 mm and inner diameter of 55 mm. The coil was applied tangentially to the scalp of patient at diaphragmatic motor cortical area, a point of optimal excitability, located 3 cm lateral to midline and 2–3 cm anterior to auricular plane [12] with face A of the coil visible from above (anticlockwise coil current) for left hemisphere stimulation and face B (clockwise coil current) for right hemisphere stimulation recording cortical MEPs responses. Surface electrodes were placed in the 7th and 8th right and left intercostal spaces respectively within the anterior axillary line, and the reference electrode on the corresponding lower rib for recording diaphragmatic cortical MEPs response contralateral to the stimulation site. A ground electrode was placed on the manubrium sterni. [20] The recording conditions utilized were: filter setting high at 3K Hz and low at 3Hz, vertical gain 0.2- 2mV/ division, and sweep speed 5 msec/ division. TMS was performed at 80% of the maximal magnetic output at the end of expiration by observing chest movement with the patients in the supine position during which diaphragmatic electromyograph was continuously monitored on the device screen (Fig 1). The angle of the coil around the stimulation site was changed until the highest MEPs response during inspiratory phase was recorded. All stimulations were repeated 5 times with time interval of 45 to 60 seconds where the best 3 recordings were selected for measurement. The values corresponded to the average of 3 stimulations. TMS testing was performed during the morning between 8–9 a.m. The following parameters were measured from central stimulation: cortical motor evoked potential latency (CMEPL) in milliseconds (ms), CMEP amplitude (CMEPA) in microvoltage (μv). Further, the diaphragmatic resting motor threshold (DRMT) was measured and expressed as percentage of magnetic stimulator output. DRMT was defined as the lowest intensity level that evoked three or more diaphragmatic CMEPs in a consecutive sequence of six stimuli after decreasing the magnetic field output in 10% steps, starting from 80% of the maximum strength. [21]

Secondly, cervical magnetic stimulation of the phrenic nerve roots in the neck was performed bilaterally. The periphery of the circular coil of the apparatus was placed 2 cm lateral to mid-line and 1–2 cm above the 5th cervical spine while the patient head slightly bent forward. The diaphragmatic peripheral motor evoked potentials (PMEPs) were recorded using the same recording electrodes setting previously discussed whereas peripheral motor evoked potential latency (PMEPL) and PMEP amplitude (PMEPA) were measured. Central motor conduction time (CMCT) was then calculated as follow: CMCT = CMEPL–PMEPL. [22]

### Statistical analysis

Quantitative data were expressed as mean ± standard deviation (SD) or median (interquartile range “IQR” = 25–75 percentile) according to the normal distribution of data while qualitative data were expressed as number and percentage (n, %). Mann—Whitney test, Kruskal—Wallis test, student t-test, Chi-square test and Spearman rho correlation were used as appropriate. Post-hoc power analysis was performed and the average calculated power was 82% considering the alpha error 0.05. ROC (receiver operating characteristic) curve and area under the curve (AUC) has been used to detect cutoff values for diaphragmatic CMEPs that could differentiate COPD from healthy individuals. All the analysis has been performed using MedCalc^®^ (version 9.2.1.0, Acacialaan 22, B-8400 Ostend, Belgium) and SPSS package (PASW Statistics for Windows, Version 22.0. Chicago: SPSS Inc.).

## Results

### Participants’ characteristics

All the baselines characteristics of COPD patients and healthy control are shown in Table 1. All the recruited patients (30 COPD patients and 17 healthy subjects) were males with no statistically significant difference between both groups regarding age, BMI, and smoking status (*p* > 0.05). However, the median smoking index was significantly higher in COPD group vs. healthy subjects (60 (45–80) vs. 20 (10–30) pack /year, *p* < 0.0001). Also 33.3% of COPD patients had associated comorbidities–namely hypertension, ischemic heart diseases, obesity and OSA–vs. 0% among healthy controls (*p* = 0.029).

**Table 1:** Demographic and baseline clinical characteristics of study population.

Character	COPD (n = 30)	Control (n = 17)	*p* value
Age (years); median (IQR)	59 (55–63)	55 (50–59.5)	0.055
**Gender**; n (%)			
Male / Female	30 (100) / 0 (0)	17 (100) / 0 (0)	1.0
BMI (Kg/mm^2^); mean ± SD	24.3 ± 4.7	22.8 ± 3.6	0.338
**Smoking history**; n (%)			
smoker / ex-smoker	14 (46.7) / 16 (53.3)	11 (64.7) / 6(35.3)	0.375
smoking index (PYI)	60 (45–80)	20 (10–30)	<0.0001*
**Comorbidities**; n (%)	10 (33.3)	0 (0)	0.029*
Hypertension / IHD / Obesity and OSA	6 (20) / 1 (3) / 3 (10)	0 (0)	
**Spirometry**			
FVC% predicted; median (IQR)	56 (50.3–66.3)	109 (98–123)	< 0.0001*
FEV_1_% predicted; median (IQR)	42.9 (29–54)	123 (112–136.5)	< 0.0001*
FEV_1_/FVC %; mean ± SD	57.6 ± 8.7	86.6 ± 8.5	< 0.0001*
**ABG**			
pH; mean ± SD	7.43 ± 0.048	NA	NA
PaO_2_ (mmHg); mean ± SD	78.43 ± 20.8		
PaCO_2_ (mmHg); mean ± SD	40.5 ± 8.9		
HCO_3_ (mmol/L); median (IQR)	25 (22–30)		
SaO_2_%; median (IQR)	96 (94.8–97.0)		
**Laboratory tests**			
FBS (mg/dl); median (IQR)	101.5 (72–111)	NA	NA
Hb (g/dl); mean ± SD	13.9 ± 1.3		
BUN (mg/dl); median (IQR)	15 (12–20)		
Cr (mg/dl); mean± SD	0.81 ± 0.24		
Na (mmol/L); median (IQR)	140 (137–144)		
K (mmol/L); mean ± SD	4.1 ± 0.35		
AST (U/L); median (IQR)	29.5 (22–41)		
ALT (U/L); median (IQR)	27.5 (20–41)		
Albumin (g/dl); median (IQR)	3.0 (2.9–3.4)		

*: Statistically significant at *p* ≤ 0.05

BMI: body mass index, OSA: obstructive sleep apnea, IHD: ischemic heart disease, PYI: pack/year index, PaO_2_: arterial partial pressure of oxygen, PaCO_2_: arterial partial pressure of carbon dioxide, HCO_3_: bicarbonate, SaO_2_: oxygen saturation, FBS: fasting blood sugar, Hb: hemoglobin, BUN: blood urea nitrogen, Cr: creatinine, Na: sodium, K: potassium, AST: aspartate transferase, ALT: alanine transferase, NA: not assessed.

Baseline FVC%, FEV_1_% and FEV_1_/FVC were significantly lower in COPD group (*p* < 0.0001) where 2 COPD patient (6.7%) had mild airway obstruction, 8 patients (26.7%) had moderate airway obstruction, 12 patients (40%) had severe airway obstruction and 8 patients (26.7%) had very severe airway obstruction according to GOLD classification.

Regarding the ABG, the median SaO_2_ was 96% (IQR = 94.8–97.0%) with mean PaO_2_ of 78.43 ± 20.8 mmHg. The mean PaCO_2_ was 40.5 ± 8.9 mmHg as 7 patients were hypercapneic (23.3%).

### Diaphragmatic neural function assessment

Both CMEPs and PMEPs of studied population are illustrated in Table 2 with demonstration example in Fig 1. Regarding CMEPs, there was a statistically significant bilateral decrease in DRMT and CMEPA in COPD group vs. healthy subjects (*p* < 0.0001). Further, there was a statistically significant increase in CMEPL bilaterally in COPD group vs. healthy subjects (*p* < 0.0001 and *p* = 0.006 for CMEPL on left and right side respectively). Left CMCT was significantly prolonged in COPD group vs. healthy subjects (*p* < 0.0001) but not for right CMCT (*p* = 0.376).

**Table 2:** Comparison between the two studied groups regarding diaphragmatic CMEP and PMEP parameters.

Parameter	COPD (n = 30)	Control (n = 17)	*p* value
**Right diaphragm conduction**			
DRMT (%); mean ± SD	66.9 ± 8.2	89.5 ± 5.2	< 0.0001*
CMEPL (ms); median (IQR)	14.4 (11.9–16.5)	11.2 (10.5–12.4)	0.006*
CMEPA (μv); median (IQR)	120 (110–140)	177 (158.3–180.0)	< 0.0001*
PMEPL (ms); mean ± SD	6.99 ± 1.05	5.4 ± 0.6	< 0.0001*
PMEPA (μv); median (IQR)	135.0 (117.0–160.0)	190 (179.5–196.3)	< 0.0001*
CMCT (ms); median (IQR)	7.7 (4.9–9.2)	5.9 (5.6–6.6)	0.376
**Left diaphragm conduction**			
DRMT (%); mean ± SD	68.6 ± 7.6	89 ± 4.4	< 0.0001*
CMEPL (ms); median (IQR)	16.8 (14.5–18.0)	10.9 (10.6–12.8)	< 0.0001*
CMEPA (μv); mean ± SD	127.1 ± 23.8	173.9 ± 34.2	< 0.0001*
PMEPL (ms); median (IQR)	7.4 (6.0–8.4)	5.1 (4.7–5.75)	< 0.0001*
PMEPA (μv); mean ± SD	147.3 ± 21.7	183.0 ± 35.9	0.001*
CMCT (ms); median (IQR)	9.3 (8.1–10.1)	6.2 (5.5–6.95)	< 0.0001*

*: Statistically significant at *p* ≤ 0.05

DRMT: diaphragmatic resting motor threshold, CMEPL: cortical motor evoked potential latency in milliseconds (ms), CMEPA: cortical motor evoked potential amplitude in microvoltage (μv), PMEPL: peripheral motor evoked potential latency, PMEPA: peripheral motor evoked potential amplitude, CMCT: central motor conduction time.

Regarding PMEPs, PMEPA on left and right were significantly decreased in COPD vs. healthy controls (*p* < 0.0001 and *p* = 0.001 respectively). Also, bilateral recorded PMEPL was significantly increased in COPD group vs. healthy subjects (*p* < 0.0001).

Table 3 shows the comparison between the right and left diaphragmatic neural function. There was statistically significant increase in CMEPL and CMCT of left vs. right diaphragm in COPD group (*p* = 0.003 and 0.001 respectively); but there was no statistically significant difference between right and left diaphragm in control group (*p* > 0.05).

**Table 3:** Comparison between right and left diaphragmatic CMEPs and PMEPs in both groups.

Parameter	COPD group (n = 30)			Control group (n = 17)		
	Right	Left	*p* value	Right	Left	*p* value
**DRMT (%)**	66.9 ± 8.2	68.6 ± 7.6	0.417	89.5 ± 5.2	89 ± 4.4	0.778
**CMEPL (ms)**	14.4 (11.9–16.5)	16.8 (14.5–18.0)	0.003*	11.2 (10.5–12.4)	10.9 (10.6–12.8)	0.783
**CMEPA (μv)**	122.8 ± 22.3	127.1 ± 23.8	0.472	177 (158.3–180.0)	173.9 ± 34.2	0.959
**PMEPL (ms)**	6.99 ± 1.05	7.4 (6.0–8.4)	0.427	5.2 (4.9–5.8)	5.1 (4.7–5.8)	0.593
**PMEPA (μv)**	138.3 ± 25.7	147.3 ± 21.7	0.147	190 (179.5–196.3)	190 (147.5–196.5)	0.986
**CMCT (ms)**	7.7 (4.9–9.2)	9.3 (8.1–10.1)	0.001*	5.9 (5.6–6.6)	6.2 (5.5–6.95)	0.629

The data are presented as mean ± SD for DRMT of both groups, CMEPA, PMEPA and PMEPL of COPD group or median (IQR) for the remaining

*: Statistically significant at *p* ≤ 0.05

DRMT: diaphragmatic resting motor threshold, CMEPL: cortical motor evoked potential latency in milliseconds (ms), CMEPA: cortical motor evoked potential amplitude in microvoltage (μv), PMEPL: peripheral motor evoked potential latency, PMEPA: peripheral motor evoked potential amplitude, CMCT: central motor conduction time.

### Correlations

**Fig 2:**
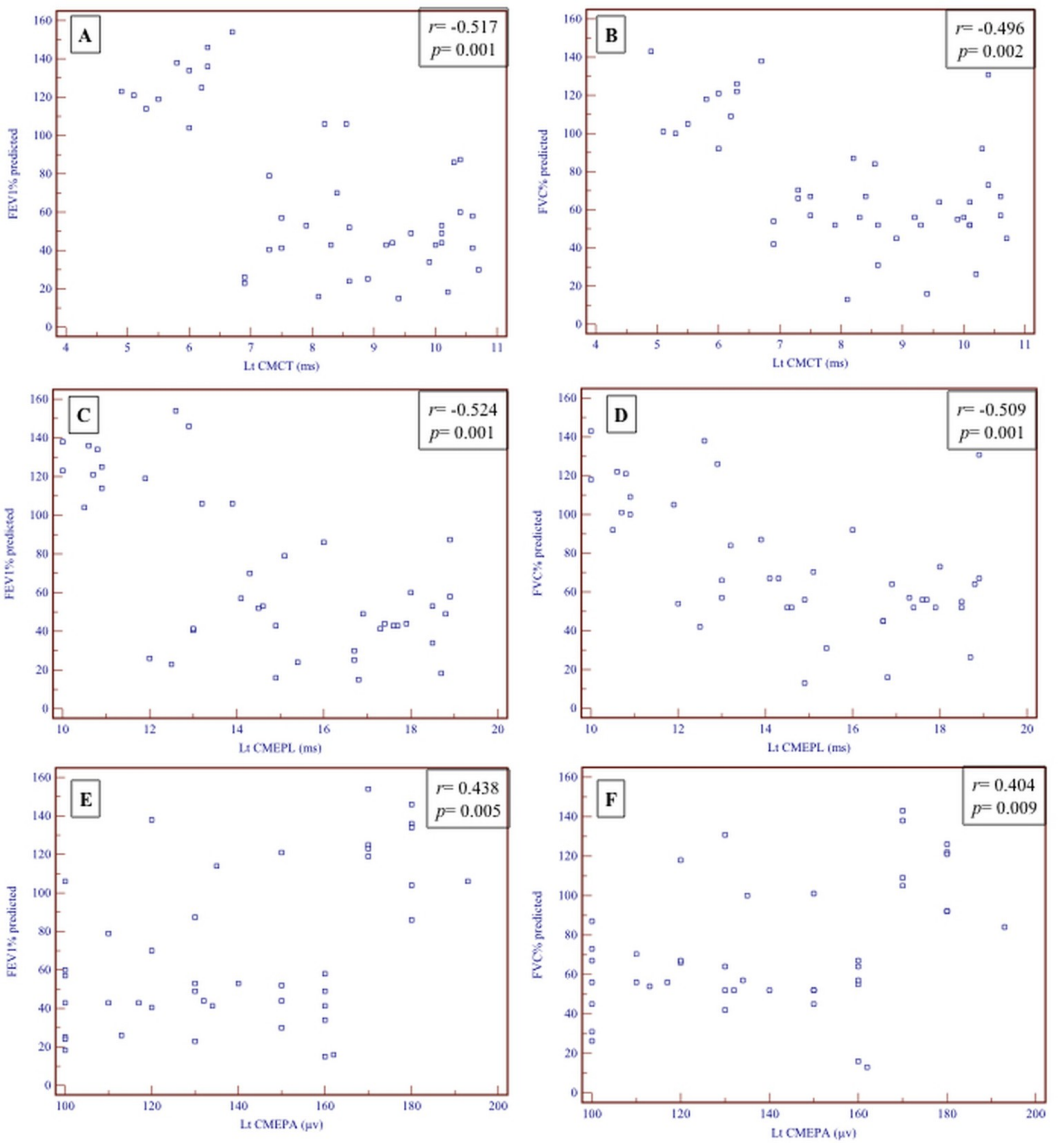
Correlations between spirometric parameters (FEV% predicted and FVC% predicted) and left CMEPs (A-F).

Left diaphragmatic CMEPL and CMCT inversely correlated with different pulmonary function parameters (i.e. FVC% predicted, FEV_1_% predicted and FEV_1_/FVC); and left diaphragmatic CMEPA positively correlated with pulmonary function parameters among the studied population (*p* < 0.01; Fig 2A–2F). However, right diaphragmatic CMCT did not correlate with pulmonary function parameters (*p* > 0.05) while right CMEPL is inversely correlated with FVC% predicted (*p* = 0.036) but neither FEV_1_% predicted or FEV_1_/FVC (*p* > 0.05) among the studied population. Both right and left diaphragmatic peripheral conduction (PMEPL and PMEPA) were positively correlated with different pulmonary function parameters (*p* < 0.01). In addition, both right and left DRMT were positivity correlated with FVC% predicted, FEV_1_% predicted and FEV_1_/FVC (*p* < 0.001).

On the other hand, there was no statistically significant association between CMEPs, PMEPs or DRMT and COPD severity according to GOLD classification (*p* > 0.05). Similarly, there was no statistically significant correlation between diaphragmatic CMEPs, PMEPs or DRMT and age, smoking status, smoking index, BMI, serum albumin or ABG parameters (*p* > 0.05).

### ROC analysis

**Fig 3:**
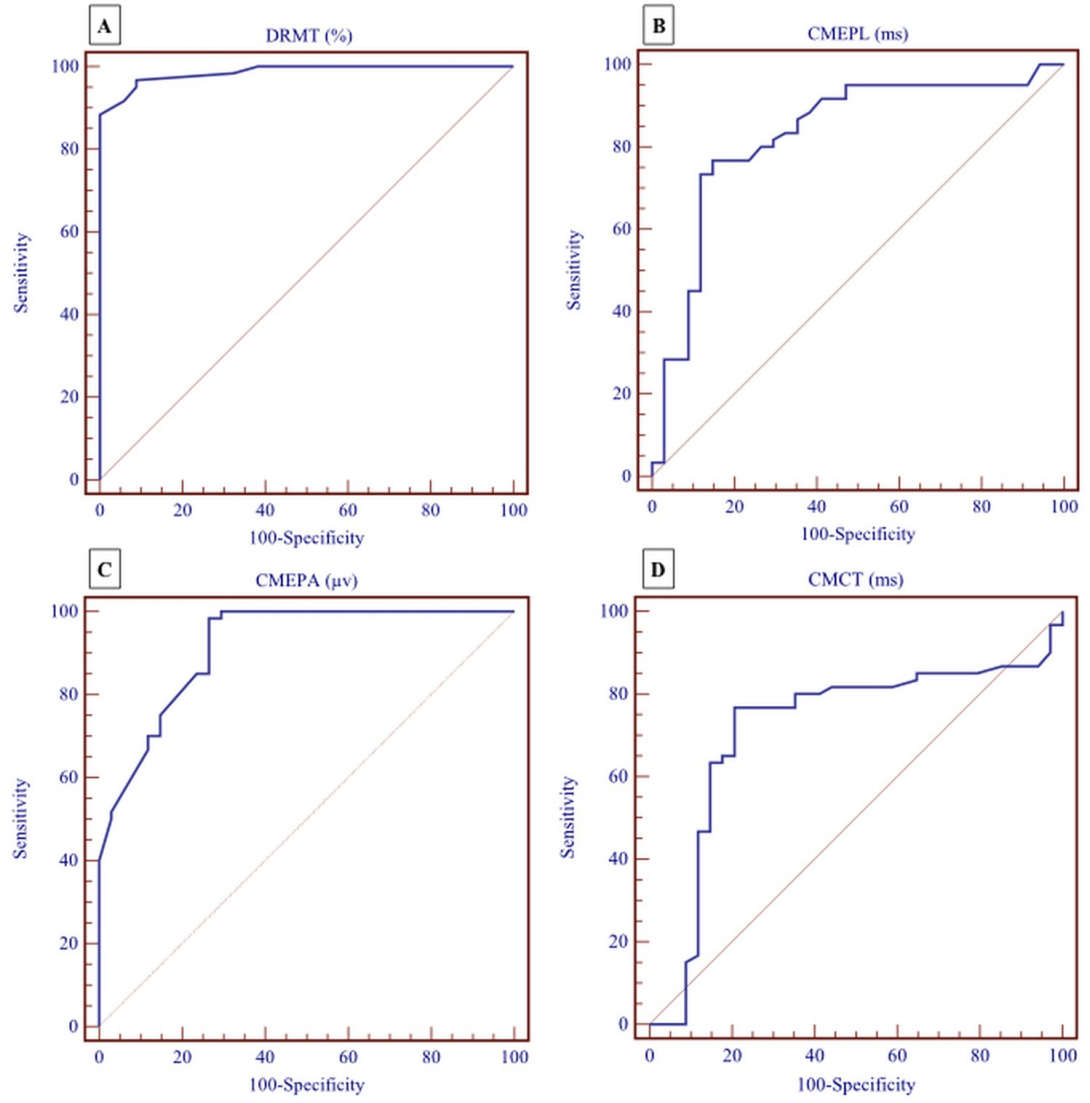
ROC analysis in COPD patients for predicting cutoff for CMEPs. (A) for DMRT% (AUC = 0.986, CI95% = 0.936–0.998, *p* = 0.0001). (B) for CMEPL (AUC = 0.828, CI95% = 0.737–0.898, *p* = 0.0001). (C) CMEPA (AUC = 0.715, CI95% = 0.612–0.803, *p* = 0.0001). (D) CMCT (AUC = 0.916, CI95% = 0.841–0.963, *p* = 0.0001).

According to ROC analysis, DRMT ≤ 80% had diagnostic accuracy of 98.6% to differentiate COPD from healthy control individuals with a sensitivity of 92% and specificity of 94% (AUC = 0.986, CI95% = 0.936–0.998, *p* = 0.0001; Fig 3A). CMEPL > 12.9 ms had diagnostic accuracy of 83% and sensitivity of 77% and specificity of 85% for differentiating COPD from healthy subjects (AUC = 0.828, CI95% = 0.737–0.898, *p* = 0.0001; Fig 3B). CMCT > 6.7 ms had diagnostic accuracy of 71.5% and sensitivity of 77% and specificity of 80% for differentiating COPD from healthy subjects (AUC = 0.715, CI95% = 0.612–0.803, *p* = 0.0001; Fig 3C). CMEPA ≤ 160 μv had 92% diagnostic accuracy, 98% sensitivity and 73.5% specificity for differentiating COPD from healthy subjects (AUC = 0.916, CI95% = 0.841–0.963, *p* = 0.0001; Fig 3D).

## Discussion

In the current study, there was a statistically significant delayed diaphragmatic CMEP and PMEP latencies as well as decreased CMEP / PMEP amplitudes and bilateral DRMT in COPD patients compared to the healthy subjects. In addition, COPD patients had a statistically significant difference between right and left central motor diaphragmatic conduction — that to our knowledge has not been discussed in previous publications — and inversely correlated with pulmonary function testing.

Hopkinson et al [14] found that diaphragmatic PMEPL was significantly longer in COPD patients than healthy controls. Similarly, Hamed et al [23] reported bilateral increase in CMEPL and CMCT in their studied COPD patients compared to healthy control group. Yu Wang et al [15] found prolonged CMCT and CMEPL evoked by TMS in patients with acute exacerbation of COPD and respiratory failure as well as prolonged PMEPL and lower PMEPA evoked by cervical magnetic stimulation. Further, El-Tantawi et al [24] found peripheral phrenic nerve conduction abnormalities in 42.5% of their studied COPD patients that did not correlate with disease severity. These results are in accordance with the current results and could be explained by increased excitation of motor cortex and corticospinal pathways to the respiratory muscles [25] and less excitability of intracortical facilitatory circuits at long interstimulus intervals in the COPD patients. [14]

Interestingly, we found significant prolonged CMEPL and CMCT of left versus right diaphragm in COPD group which inversely correlated with FEV_1_% and FVC% predicted but not ABG parameters. This denotes that there is heterogeneity in affection of respiratory muscles which is in accordance with disease heterogeneity. [26] Moreover, increased inspiratory load of respiratory muscles has been associated with significant activation of several motor cortical areas as demonstrated by increased regional cerebral blood flow using positron emission tomography [27] which could be affected asymmetrically. More recently, Dodd et al [28] demonstrated by magnetic resonance imaging techniques that generalized functional activation of resting-state networks in COPD patients compared to controls.

On the other hand, right and left DMRT were insignificantly different despite being diminished in COPD patients compared to healthy subjects that correlated positively with different pulmonary function parameters. Hopkinson et al [14] and Hamed et al [23] found that DRMT was significantly lower in stable COPD than healthy controls. Also, Hopkinson et al [25] in another study found that DRMT did not change after the use of non-invasive ventilation for stable COPD either in short or long term. The stability of DRMT could represent cortical metaplasticity (i.e. change in the capacity of plasticity expression secondary to prior exposure) [29] which could be due to load capacity imbalance of respiratory muscle pump in COPD patients. [25]

Further, we proposed cut-off point for CMEPs (i.e. CMEPL, CMCT, CMEPA, and DRMT) that had good diagnostic accuracy and sensitivity for predicting corticospinal pathway affection in case of COPD men patients. Lissens [7] demonstrated values for diaphragmatic CMEPs in 10 healthy man only, which were close to the values recorded in our healthy subjects. However, to our knowledge, there are no specific values proposed to date that could be reference for CMEPs responses in COPD. We suppose that these values might be considered as reference, however, further studies with larger population should be considered to confirm these values.

Diaphragmatic dysfunction is strongly correlated with FEV_1_ in COPD [30] and correlated with the perception of dyspnea among this group of patients. [31] Coticospinal pathway dysfunction could be another factor for the development of diaphragmatic dysfunction in COPD patients. Accordingly its evaluation could help in the personalization of COPD management especially pulmonary rehabilitation programs. Chun et al found significant improvement of diaphragmatic motility after pulmonary rehabilitation using sonography. [32]

Further, assessment of diaphragmatic corticospinal pathway could be of value in evaluation noninvasive ventilation use in stable severe and very severe COPD. [33] This has been demonstrated by Hopkinson et al [25] who found that the excitability of the corticospinal pathway to the diaphragm was reduced in 6 COPD patients after acute noninvasive ventilation use. This could be explained by the fact that noninvasive ventilation reduced inspiratory muscles loads [33] through reduces the cortical motor areas excitability supplying the respiratory muscles especially the diaphragm. [34] Accordingly, TMS could be a good applicable tool for the evaluation of central and peripheral diaphragmatic neural pathway which may affect the future management of COPD patients.

The current study has some limitations. Firstly, we studied only the diaphragm as the main respiratory muscle and we did not study the intercostals or abdominal muscles. This could be because the cortical area for the diaphragm has been previously validated in healthy man [12,13] rather than other respiratory muscles. Secondly, we used surface electrodes for diaphragm CMEPs recording and we did not use diaphragm needle electromyography. However, intercostal surface electrodes have been validated previously [20] and needle electromyography is more invasive and could be associated with complications as pneumothorax. Thirdly, we did not study the diaphragmatic CMEPs response at different intervals of time of at maximal inspiratory efforts in COPD patients. However, Sharshar et al [35] studied before the response to cortical stimulations at different points of time or inspiratory efforts in healthy men and they concluded that cortical motor control of diaphragm is identical during different inspiratory tasks. Fourthly, the studied population were only males with different severity of pulmonary function. COPD is more prevalent in men worldwide that is caused mainly by smoking [36,37] and it was not surprising to have more prevalence of COPD in men in our community. Further, COPD is a complex heterogeneous disease with various severity of airway obstruction that is associated with respiratory muscle dysfunction, contribute to their disease prognosis irrespective of the lung function. [38] Lastly, the studied population was relatively small; however, the post-hoc power analysis was 82%, CI 95% was narrow with AUC > 0.8 in most of tested CMEPs which supports the relevance of the analyzed data and its good power.

## Conclusions

Central cortico-diaphragmatic motor system is affected in COPD patients with heterogeneity of both sides that is correlated with airway obstruction but not with COPD severity or ABG changes. Moreover, the current study suggests that if the CMEPs shows prolonged latency and conduction time, this might predict diaphragmatic corticospinal affection in COPD patients. These findings could be a step for future studies directed towards the evaluation of the diaphragm in COPD especially after various therapeutic interventions using a noninvasive tool as the TMS.
